# A Lesson in Comparative Zoology

**Published:** 1882-10

**Authors:** Hubbard W. Mitchell


### ART III.—A LESSON IN COMPARATIVE ZOOLOGY.

BY HUBBARD W. MITCHELL, M.D.

Whoever enters for the first time into the extensive field of Zoology, will see what appears to be a very wide difference among the various members composing the Animal Kingdom, and the theory so strenuously advocated by Agassiz and others, that each species was the result of a special creative act, might seem, upon first sight, a very plausible one. If we could accept this theory, then each species of animals could have sprung into existence fully formed, as a special act of creation, and the species would have remained immutable. The creation of species could have been at the pleasure of the caprice of the creative power, and a fish, reptile, mammal, insect, or mollusk could have been formed in this, or in any other order, as it pleased that power.

But such a theory is contrary to the teachings of modern scientific research. We can now see a regular and harmonious sequence in the order of creation, an order beginning at the lowest forms of life, and proceeding by slow, and regular, and gradual steps, up to the higher. This evolution of the living forms shows no abruptness, but a series of successive changes, where one form has, during considerable lapses of time, given place to another and modified form. On this principle of evolution, can be explained not only every form of life on earth now, but also all the extinct forms that have existed in past geological ages, and whose fossil remains are found in the various strata of the earth.

The different species of animals, both fossil and now living, have been evolved, the higher from the lower, during long lapsse of time, and modified by climate influences, and natural selection, or the "survival of the fittest." It might be interesting, if it were possible, to inquire how these changes have been carried on, so as to form, for instance, a lizard from a worm, an animal from a lizard, or lastly, man from an animal.

That such changes have taken place is beyond doubt. It is impossible to believe otherwise, when we follow the orderly series from a worm upt o man.

Naturalists tell us that the first form of life was a cell—a cell or body containing within itself the power to divide and subdivide, and in this way to give rise to other cells. In other words, to multiply by fission and growth. Now, if this is so, and I think we may safely assume that it is, let us apply this principle, and see if we can, by its aid, study the laws of evolution and ascertain how different forms of life came into existence.

A simple cell, then, was a living body, having, we may say, a round or an oval shape.

This cell by fission produced, we will assume, a dozen other cells.

Now, if this dozen cells were placed end to end, we would have a very respectable worm.

In the course of time changes of geological character took place, and made a higher grade of life possible, and the step to a lizard by the growth or addition of cells was an orderly one. From this primitive creature the higher order of saurians were undoubtedly evolved, until later, in the Triassic and the Liassic periods, the huge Plesiosaurus and the Ichthyosaurus were gradually developed. The step to a bird from these lizards was soon taken, a clearer atmosphere making it possible for them to live, and the curious Pterodactyle was evolved. Later on in geological ages, the Pterodactyles were slowly modified, their long fingers were shortened, and the bat-like web between them became covered with feathers and formed wings—modified cells still—and true birds came into existence.

In the Cretaceous period we find another order of animals retaining many saurian qualities, but pointing towards quadrupeds. The curious Iguanodon, the transition animal from the Ichthyosaurus to the next higher order, the Megalosaurus. Following our same principle, we have but to shorten the lizard's tail to increase the size of his head, body and limbs by adding a few more cells, or taking some of them away to form either of these animals. From the Megalosaurus we can pass by slow evolving steps to the mastodon, the rhinoceros, the hippopotamus, the tapir, and the elephant.

If we go back to the Megalosaurus we shall see that quadrupedal life modified from him gave rise slowly to the Felidæ, such as the lions, tigers, and panthers; the Canidæ, such as the wolf, jackal, and dog, and the hyena. From the Anoplotherium came the horse, ass, zebra, and, probably, also the different species of deer.

If now we again modify our primitive lizard, shorten his body and fore legs, and tilt him up on one end, we have the kangaroo. By modifying steps again, we can abridge the enormous tail of this animal, make hands of his fore paws, and by slowly evolving steps the monkey emerges. By higher steps, and a modification of parts, a greater development of limbs into more perfect shapes, and a larger growth of the cerebral structures, and man comes as the last and highest order of evolution.

In this long process of evolution, continuing through vast periods of time, an orderly sequence of the forms of life has been observed from the lowest to the highest. Climatic changes, geological changes, have been all-powerful in producing these changes of structure in animal forms.

I have left out of consideration a great number of animals, birds, fishes, and reptiles that would more clearly show this sequential evolution of species. Indeed, in a brief sketch like this, only the *plan* can be alluded to ; a volume would be needed to describe the process, if description were possible. But perhaps enough has been said to show that animal life could easily have begun in a single cell, and that during *immense geological ages*, modifying influences of climate and of the atmosphere, the emergence of the land from the sea, and the introduction of vegetation in the carboniferous age, the further influence of natural selection, have been sufficiently powerful to produce one after another the animal forms that have lived and flourished on the earth.

We see now that all the animal tissues are made up of cells, modified so as to produce bone, tendon, nerve, muscle, and so on, and in the vegetable world, the soft petals of flowers, and the hard sharp thorns of some plants are *cells* modified to form these various parts. So, in the past, a few cells made a worm, a few more added and modified give the worm feet and a distinct head, then he became a saurian. More cells added to his body and fewer put into his tail, and he forms a *quadruped*, and so *he* goes on modifying, new forms arise, as new modes of life become possible, and thus higher orders are evolved. Meantime geological changes are taking place, and some of the earlier forms now find these changes incompatible with their life, and they die.

I believe that if we could find *all* the forms of life that have ever existed on the earth, from our worm up to man, we should see a gradual development upward, and that there would be no "missing link."

In glancing back over this long array of regular, orderly evolution of life, which has at last culminated in man, Figuier asks if there may not arise in the future some being that shall be as superior to man as man is superior to the animals below him.

Perhaps some of the later human species can answer his question.

Having entered the wide field of Zoology, we see before us a multitude of animals of different forms, sizes, colors and habits. At first view there does not seem to be the least similarity between any two of them. All appear totally different and distinct.

Here the naturalist and the comparative anatomist steps in, and begins to study this great mass of animal life, and see upon what plan, if any, it is formed.

He begins by comparison. He compares one form with another, and sees if any two or more animals have any qualities in common. If so, he assigns them to some order and species, and in this way he simplifies their study.

Let us apply this principle of comparsion, in practice. Suppose we see together for instance the following:

A Pig, Tapir, Peccary, Elephant . . . . . . . 1
A Lion, Tiger, Panther, Cat . . . . . . . 2
A Dog, Wolf, Fox, Jackal . . . . . . . . 3
A Goat, Deer, Ox, Sheep, Camel . . . . . 4
A Horse, Ass, Zebra . . . . . . . . 5

How shall we compare them, in order to find features common to any two or more of them?

Perhaps the feature that strikes us first would be the horns in the ox and the deer. Can we associate these with any of the others?

If we examine the teeth of all the animals in the first, second and third lines, except the elephant, we shall find them sharp and cutting, with long canine or prehensile teeth, admirably adapted for seizing and holding living prey, and for cutting and tearing it. We should, therefore, call these animals carnivorous. If we examined the teeth of the animals in the fourth and fifth lines, we shall see that they have large flat surfaces well adapted to chewing and grinding, and as they live on vegeteble substances, we should call these animals herbivorous.

Comparing still further, we find that in the horse and the ox, both herbivoræ, the horse has incisor teeth in the upper and the lower jaw, while in the ox there are incisiors in the lower jaw only, and this arrangement of teeth has a direct relation to the different structures of the stomach in the two animals, the ox having a complicated stomach with four pouches adapted to a mode of digestion by which the food is prepared for a second mode of mastication. This order of animals, those in the fourth line, are called ruminants.

The animals in the fifth line have simple stomachs, and do not ruminate.

If we again compare the ox, this time with the deer, we shall find that while both are herbivorous ruminants, and both have horns, in the ox the horn is hollow and remains firmly attached to the animal's head through life, in the deer it is solid and is shed every year.

Upon looking further we find that the pig, tapir and peccary, carnivorous animals, have a cloven foot like the herbivorous animals in the fourth line, and those last again, are different from the herbivorous animals in the fifth line, which have a solid hoof or foot.

These comparative studies will enable us to classify animals into different orders and species.

Naturalists tell us that certain animals sprang from a common stock, and the differences between them are the results of modifications of structure gradually occurring during long periods of time. Thus the pig has a stout rounded body and a mobile nose. The peccary is a step higher. Then in the tapir, the body is about the same, but the nose is decidedly more developed. The rhinoceros comes in next with the same form of body, and his nose modified and turned upwards into one or two horns, and lastly the elephant, with his long mobile nose or trunk, is the highest expression of this common stock.

The order Felidæ, or cats, are almost precisely alike, the differences being chiefly of size, color, and growth of hair. The lion, tiger, panther, leopard, puma, and cat certainly sprang from a common stock. The domestic cat, perhaps, having the highest development from the fact of its ability to rotate the radius upon the ulna, a motion the larger cats do not have. We see the grace of this motion when our cats play with a mouse or a ball of yarn.

The same fact is seen in the Canidæ, or dogs. They came from a common stock also. The wolf, jackal, dog and fox are of the same species, and have the same structure.

In the Gramnivorous Ruminants, the origin from a common stock is apparent. The sheep, with its woolly coat and curved horn, undergoes a slight modification, and we have the goat, also with a curved horn and woolly hair. A further development gives us the deer, and further still the ox. Then comes the camel, and lastly the camelopard, or giraffe.

As an interesting instance of the structure of animals conforming to their modes of life, the giraffe may be mentioned. This animal feeds upon the foilage of trees. Upon his head he has tufted sensitive horns that he may *feel* his food as he passes along. His elongated neck enables him to reach it easily, and in order that he may be on the lookout for his enemies, the crouching lion being his most dreaded enemy, while his head is so high above the ground, his eyes are set so that he can see above, below, forwards or behind without moving his head.

The complicated digestive apparatus of ruminants has some relation to their methods of escape from danger. As their food consists principally of vegetable substances, little nutritious and demanded in large quantities, and as they are in turn food for the ferocious carnivorous animals, their only means of safety is in flight, while mastication is a work of time.

They are, therefore, obliged to graze rapidly, fill their large stomach reservoir with unchewed food, and then retire to a place of safety, where they can re-masticate it at leisure.

The horse, ass, zebra and other animals of this order sprang also from a common stock.

We have now seen that by comparing animals with each other we are able to classify them into orders and species, each order and species having some distinctive character entitling them to a fixed and permanent place in zoological classes.

If now we examine all the forms of animal life on the earth, the fauna, or the animal kingdom, we shall find that they can be comprised under *four* great heads—namely, Vertebrates, Articulates, Mollusks, Radiates.

It is with the *first* of these only that this paper will deal.

As we saw that animals were classified under several heads by examining them externally and internally to a limited extent, and this classification gave us Carnivoræ, Herbivoræ, etc., so now if we examine this immense order of Vertebrates by the study of their bony frame-work, or *osteology*, we shall find that all, from an eel up to man, are constructed upon one single plan.

Beginning with man and descending in the scale, we will briefly examine this bony frame-work, or skeleton, and see what this plan is upon which such a vast part of the animal world has been constructed.

First we find a skull, rounded in shape, hollow, having apertures for the eyes, mouth, etc., jaws set with teeth, and articulated upon the first of a series of bones, or vertebræ. These vertebræ joined together by ligaments so as to form a continuous column, or backbone, mark the distinctive features of the whole order—viz., Vertebrates.

From the upper part of this spinal column are given off twelve pairs of ribs, which, nearly meeting in front, form a bony cage containing the heart and lungs.

The uppper extremities are formed by a scapula and a clavicle, a humerus, radius, ulna, carpus, metacarpus and phalanges.

At the lower end of the spinal column is the pelvis, from which is given off the femur, the tibia and fibula, the tarsus, metatarsus and phalanges to form the lower extremities.

This briefly is man.

In the Simiidæ, or monkeys, the facial angle is a little greater, the humerus, the radius and ulna a little longer, the tarsus slightly modified, and in some species the vertebræ are extended into a tail. These are monkeys modified a little from man.

In the Felidæ and the Canidæ, the facial angle is still greater, the teeth sharp and cutting, with well developed canines, the upright position is changed to a horizontal posture, the upper extremities are now fore limbs, bones modified in shape, species mostly digitigrade. The bears are all plantigrade.

General plan same as man.

In the remaining members of the Carnivoræ, as the sea lion, seal and walrus, the fore limbs are modified into paddles, while the hind limbs are less developed. In the walrus the lower incisors and canines are absent, while the upper incisors are elongated into tusks.

General plan same as man.

In the Bovidæ, beginning with the camels and running through the llama, giraffe, ox, bison, yak, goat, zebu, gazelle, gemsbok, sheep, antelope and deer, we have an elongated skull, modified bones of the fore and hind limbs, and a modification also of the carpus and metacarpus, the tarsus and metatarsus, whereby the foot is cloven, owing to a consolidation of some of these bones. In some of the members of this order we have the horn, either deciduous or permanent, and in all the cauda. In some, also, we have a longer scapular and longer spinous processes. This affords greater attachments to the nuchal ligaments and muscles for those animals with heavy heads and horns. In the giraffe this is especially noticeable. The fore shoulders are so high that it makes the fore legs seem higher than the hind legs. This is due to the lengthened scapula and spinous processes. The heads of the femur and humerus are on the same level.

In all other respects, the general plan is the same as man.

In the Cetacea, as the porpoise, dolphin and whale, the skeleton is the same, except the hind limbs are modified into complete or rudimentary fins.

In the extensive orders of Insectivora, Rodentia, Edentata and Marsupalia, the general plan is that of the higher animals, and of man, modified to meet the requirements of its mode of life.

In the enormous class of Aves, or birds, beginning with the robin and ending with the penguin, we have the elongated skull ending in a beak or bill, light porous bones, an extensive sternum, clavicles united, to support the wings, various modifications of the humerus, radius, ulna, and the carpus, metacarpus and the phalanges, as in the bats, etc.

In all the species of birds, the general plan is the same as man.

The same is true of the class Reptilia and Amphibia. In some of the snakes the legs are rudimentary, in others the ribs serve as legs. In the sharks the fins take the place of the fore and hind limbs.

In the class Pisces, or fishes, through the whole series down to the eel, we have the skull, vertebræ and ribs, and the bones of the fore and hind limbs are more or less perfectly represented by the fins. The pelvis in fishes is rudimentary, or wanting.

This brief sketch of the Vertebrata, from man to the lowly eel, shows that the whole sub-kingdom is formed upon a single plan, and that any variations are but simple modifications designed to meet some special requirement. The study of osteology shows us that in this long line of animals—the Vertebrates—the distinctive feature is the spinal column, and it is astonishing, as well as interesting, to observe how little difference there really is between the backbones of any two members of the whole series. The chief differences are in the structure of the head and limbs.

If we compare the anterior limbs of some of the lower animals with the arms of man, we shall see that bone for bone is present, from the humerus to the phalanges. For instance, take the anterior limb of, say, an ape, a bat, a dog, a mole, a deer, a whale, a seal, a tortoise, a fish, or a bird. In each we can distinctly trace the humerus, the radius and ulna, the small bones of the carpus and the metacarpus, and the phalanges.

The differences of shape in each animal are simply modifications of the same bone to adapt it to its individual mode of life.

The intelligence of animals, no doubt, resides in the brain, and it is believed that the amount of the reasoning power, or faculty, is in some way proportioned to the quantity of *gray matter* of the brain.

In connection with the reasoning power of animals, depending on brain action, may be mentioned a provision of nature for the safety, which—although it has nothing to do with reason—acts in connection with it.

This is the *mimicry* of animals, or the adaptation of the color of an animal to that of surrounding objects in its native wilds.

Nature excites our wonder by her wisdom of this curious and interesting provision.

Thus the tiger, so beautifully decorated with black stripes upon a ground of reddish-yellow fur, tending to white below, living in the long jungle grass of Southern Asia, with the color of which its stripes so closely assimilate, it is impossible for an unpracticed eye to discern it even at a short distance. This mimicry not only protects the animal's safety, but enables it to steal unseen upon its unsuspecting prey.

The uniform dun color of the puma gives it a mimicry for its safety and attack, while crouching upon the branches of trees.

The dark circular spots upon the skin of the leopard give it a mimicry that utterly deceives, as it conceals itself among the leaves of bushes and trees.

The giraffe has, perhaps, the most astonishing mimicry of any animal. Inhabiting as it does the forests of Africa and feeding upon the boughs of trees, its great size makes it a most conspicuous object. Its most dreaded enemies are the stealthy lion and man. In the regions it most frequents are many dead and blasted trunks of trees, and its mimicry is such that the most practiced eye has failed to distinguish a tree trunk from a giraffe, or a giraffe from a tree trunk.

Reliable accounts have reached us where lions have gazed long and earnestly at a motionless giraffe, and being in doubt whether it was a tree or not, have actually turned and skulked away.

Mimicry exists more or less throughout the animal kingdom. Trusting that what has been said above will awaken a proper interest in this wide and extensive subject, and lead others to enter the field where there is so much room to labor, and where so little has been done, I will close this brief lesson in comparative zoology.

